# Does posture explain the kinematic differences in a grounded running gait between male and female Svalbard rock ptarmigan (*Lagopus muta hyperborea*) moving on snow?

**DOI:** 10.1007/s00300-021-02872-x

**Published:** 2021-05-05

**Authors:** Andres Marmol-Guijarro, Robert Nudds, Lars Folkow, John Lees, Jonathan Codd

**Affiliations:** 1 School of Biological Sciences, Faculty of Biology, Medicine & Health, University of Manchester, Manchester, UK; 2 Department of Arctic and Marine Biology, University of Tromsø—The Arctic University of Norway, Tromsø, Norway; 3 Department of Physics, Chemistry and Biology, Linköping University, Linköping, Östergötland Sweden

**Keywords:** Arctic, Sexual dimorphism, Biomechanics, Substrate

## Abstract

The majority of locomotor research is conducted on treadmills and few studies attempt to understand the differences between this and animals moving in the wild. For example, animals may adjust their gait kinematics or limb posture, to a more compliant limb, to increase stability of locomotion to prevent limb failure or falling on different substrates. Here, using video recordings, we compared locomotor parameters (speed range, stride length, stride frequency, stance duration, swing duration and duty factor) of female Svalbard rock ptarmigan (*Lagopus muta hyperborea*) moving in the wild over snow to previous treadmill-based research. We also compared the absolute and body size (body mass and limb length)-corrected values of kinematic parameters to published data from males to look for any sex differences across walking and grounded running gaits. Our findings indicate that the kinematics of locomotion are largely conserved between the field and laboratory in that none of the female gaits were drastically affected by moving over snow, except for a prolonged swing phase at very slow walking speeds, likely due to toe dragging. Comparisons between the sexes indicate that the differences observed during a walking gait are likely due to body size. However, sexual dimorphism in body size could not explain the disparate grounded running kinematics of the female and male ptarmigan, which might be linked to a more crouched posture in females. Our findings provide insight into how males and females moving in situ may use different strategies to alleviate the effects of a variable substrate.

**Supplementary Information:**

The online version contains supplementary material available at 10.1007/s00300-021-02872-x.

## Introduction

Although terrestrial animals moving in the real world almost never experience stable substrate conditions, locomotion studies are overwhelmingly conducted through treadmill experiments (e.g. Abourachid 2000; Abourachid and Renous 2000; Rubenson et al. 2004; Nudds et al. 2010; Tickle et al. 2010; Watson et al. 2011). Attempts have been made to understand the influence of substrates, including the incorporation of additional factors affecting locomotion such as substrate friction coefficients with the feet (Cappellini et al. 2010; Clark and Higham 2011), substrate compliance (Lejeune et al. 1998) and/or substrate irregularities (Daley et al. 2006, 2007; Daley and Biewener 2006; Birn-Jeffery and Daley 2012). It is only by direct comparison, however, that the real effect(s) of substrate variations can be ascertained. For example, reductions in stride frequency (${f}_{\mathrm{stride}}$) and increases in stride length (${l}_{\mathrm{stride}}$) for a given speed (*U*) have both been reported in rodents and humans when moving over ground compared to on treadmills (Herbin et al. 2007; Riley et al. 2008). When running humans also experienced greater moments acting around the limb joints due to increased ground reaction forces (GRF) caused by the relatively stiffer natural ground compared to the more compliant treadmill belt (Riley et al. 2008). On slippery substrates, the low frictional coefficient between the substrate and the feet makes it challenging to attain enough GRF, increasing the risk of missing the step and falling (Cappellini et al. 2010; Clark and Higham 2011). This risk is mitigated by ensuring that the centre of mass (COM) remains close to or directly above the supporting limb via reductions in the retraction angle of the limb, resulting in smaller ${l}_{\mathrm{stride}}$ in avian (Clark and Higham 2011) and non-avian bipeds (Cappellini et al. 2010). Unexpected changes in substrate can trigger the proprioceptive responses of limb muscles that alter limb posture to facilitate either recovery of energy through elastic recoil or to absorb/produce mechanical energy depending on how the foot lands on the ground (Daley and Biewener 2006, 2011; Daley et al. 2007). Intrinsic factors such as differences between sexes can also have significant effects on the metabolic cost and kinematics of locomotion (Lees et al. 2012a). Generally, however, data documenting sex-specific locomotor characteristics are lacking, particularly for species moving over natural ground in situ in their own habitats.

A broad range of experimental locomotor data demonstrates that limb posture is a major factor influencing limb biomechanics, locomotor energetics and the effectiveness of energy saving mechanisms (Reilly et al. 2007). Defining stability as “the ability of a system to return to a steady-state, periodic gait after a perturbation” following Full et al. (2002), comparative studies suggest that the more crouched-postured species are inherently more stable and manoeuvrable during locomotion compared to species with relatively more upright and stiffer limbs (Gatesy and Biewener 1991; Daley and Usherwood 2010). Greater stability is a result of proportionally longer stance times (${t}_{\mathrm{stance}}$) that alleviate and redistribute GRF acting over the limb (Alexander and Jayes 1983; Gatesy and Biewener 1991; Andrada et al. 2013). Often, crouched-postured species also use bouncing gaits with duty factors (*DF*) greater than 0.5, meaning they grounded run with no aerial phase which is thought to improve stability (Alexander and Jayes 1983; Gatesy and Biewener 1991; Abourachid and Renous 2000; Daley and Usherwood 2010; Andrada et al. 2013); however, empirical data to support this are lacking. It is possible that a more stable species with a more crouched posture would experience fewer changes in kinematics parameters when walking over variable substrates relatively to over solid ground.

Structural (Biewener 1989), physiological (Usherwood 2013) and biomechanical (Daley and Usherwood 2010) factors all influence the limb posture of animals. The scaling of morphological components (i.e. muscles and bones) and related biomechanical concepts (i.e. stress and strain) are thought to drive the phenomenon whereby smaller species tend to have a more crouched posture and larger species have more upright limbs (Jenkins 1971; Alexander and Jayes 1983; Biewener 1989; Gatesy and Biewener 1991). Relatively small species have proportionally longer (i.e. the sum of all the leg segments) crouched limbs that move over a greater range of angles during the stance phase (${t}_{\mathrm{stance}}$) (Gatesy and Biewener 1991), allowing for higher duty factors (*DF*) that optimise the volume of active muscle to power locomotion (Usherwood 2013). These generalities hold true across a number of distantly related taxa, including members of the mammalian families of canids, felids, mustelids and ceratomorphids (Alexander and Jayes 1983; Biewener 1989), and avian representatives of the galliformes and palaeognates (Gatesy and Biewener 1991). However, any scaling effects are complex and not always uniform for phylogenetically close taxa or at an intraspecific level. For example, in felids, limb posture remains uniform among 9 species despite differing in body mass up to 41-fold (Day and Jayne 2007). Conversely, in cercopithecine monkeys of comparable body size, Polk (2002) found that extended limb postures were often associated with longer limb segments. Interestingly, size-dependent shifts in posture towards increased erectness with increasing body size is not always the case. In fact, among nine species of varanid lizards, including the small *Varanus brevicauda* (7.6 g) and the large *Varanus komodoensis* (40 kg), the largest species of the clade compensate for larger stresses of being larger by increasing the muscle mass and physiological cross-sectional area (PCSA; which is positively correlated with the muscle force output) of the muscles at the hindlimb joints (Dick and Clemente 2016).

The Svalbard rock ptarmigan (*Lagopus muta hyperborea*) is an ideal model species for examining limb posture during locomotion as its locomotor performance has been extensively studied, including comparative work spanning the laboratory and field (Marmol-Guijarro et al. 2019). Svalbard ptarmigan are also interesting as treadmill-based studies in these birds demonstrate that there is strong sexual selection towards improved locomotor performance in males compared to females (Lees et al. 2012a). Males use walking, grounded, and aerial running gaits and are able to make energy savings upon the transition to aerial running (Nudds et al. 2011). In contrast, females do not use an aerial running gait and their locomotion is *circa* 34% per kg more metabolically expensive than males moving at the same speed (Lees et al. 2012a). These differences in male and female performance are thought to result from sexual selection acting on the males, likely related to agonistic encounters with other males to secure a breeding territory (Unander and Steen 1985). Comparable studies analysing walking kinematics and limb posture in birds have only been made in leghorn chickens on treadmills (*Gallus gallus domesticus*)*.* When comparing limb posture between sexes of large and bantam chickens, postural sex differences are only found in the larger variety, with females having a more erect limb posture despite being smaller than males, relatively, whereas no differences were found between both sexes of the small variety (Rose et al. 2015, 2016b). Whether postural changes become apparent at the onset of maturity in the leghorns is unclear; however, evidence of this has been reported in the Chacma baboons *Papio hamadryas ursinus*, where adult males walk with more upright limbs than comparatively lighter females and younglings (Patel et al. 2013). In leghorns, males experience proportionally larger increases in limb muscle mass than females (Rose et al. 2016a). This sex-specific difference may cause postural changes particularly in males at the onset of maturity. Similar to leghorns, the disparities in locomotor performance between male and female Svalbard ptarmigan are less evident in juveniles, which have comparable kinematics and energetics to adults (Lees et al. 2012b). Adult male ptarmigan, however, are 6% heavier and possess longer limbs (172.7 mm) than adult females (153.2 mm) (Steen and Unander 1985; Lees et al. 2012a) which might lead to potential differences in limb posture.

Recently we compared locomotor kinematics in male Svalbard rock ptarmigan when moving on a treadmill and in the wild under natural ground conditions (Marmol-Guijarro et al. 2019). Overall, the kinematics of locomotion were conserved when the birds were walking slowly and aerial running; however, important differences were found during grounded running. When moving on snow with a grounded running gait, the limb kinematics of male ptarmigan demonstrated a reduction in ${l}_{\mathrm{stride}}$ and an increase in ${f}_{\mathrm{stride}}$, presumably because of an early retraction of the limb moments before the foot lands on the ground. By making this adjustment, males would be capable of stabilising their body, if needed, after an unexpected perturbation caused by the snowy substrate (Seyfarth et al. 2003; Daley and Biewener 2006; Daley et al. 2006). Females in the wild would be affected by snow as well; however, it is unclear if they will alter kinematic adjustments in response to the uneven and highly variable snowy substrate, irrespective of their gait.

Redressing the lack of comparable wild data for female ptarmigan was the principal aim of our current research. Accordingly, here, we compared the gait kinematics of female Svalbard rock ptarmigan moving in situ over natural snowy substrates to previous research on females moving on treadmills (Lees et al. 2012a), to determine how substrate affects their locomotion. We also examined how comparable the effects of snow are on the limb kinematics between female ptarmigan with data previously published for wild male ptarmigan by Marmol-Guijarro et al. (2019). Given that the males of this species are larger than the females, strategies to negotiate movement over snow would be dynamically comparable if the spatial (i.e. ${l}_{\mathrm{stride}}$) and temporal (i.e. ${f}_{\mathrm{stride}}$, ${t}_{\mathrm{stance}}$ and ${t}_{\mathrm{swing}}$) kinematic parameters of the limb are scalable after accounting for body size (Alexander and Jayes 1983). As both sexes must move over the same snowy substrates, we hypothesise that walking and grounded running in wild female ptarmigan should be dynamically similar to the kinematics of the wild males. In other words, females should use shorter but faster strides, with reduced periods of absolute ${t}_{\mathrm{stance}}$ to support the body within each stride (Alexander and Jayes 1983; Gatesy and Biewener 1991; Abourachid and Renous 2000), all of them scalable to the limb kinematics of the males.

## Material and methods

The terrestrial locomotion of wild female Svalbard ptarmigan *(L. muta hyperborea*, *n* = 58) moving over snow in Adventdalen and adjacent side valleys (78° 18′ 13″ N, 15° 38′ 30″ E) in Spitzbergen on the Svalbard archipelago was examined through video recordings during spring (April–May) in 2017, 2018 and 2019, coinciding with the beginning of the breeding season. During this period the midnight sun was already present, the birds are at their lowest seasonal body weight and females are not egg bearing (Steen and Unander 1985; Stokkan et al. 1986). Females were distinguished from males by the presence of a relatively thin dark eye stripe, their reduced supra-orbital red combs and by their quieter ‘kee-ah kee-ah’ calls (as opposed to the thicker eye stripe, pronounced combs and characteristic loud ‘aarr-aa-ka-ka’ calls in males). Each video recording consisted of filming a bird moving parallel across the camera frame (held at a fixed height and distance from the subject during recording) over level ground, at either 25 frames per second (fps) with a SONY® Handycam HDR-XR250 during the 2017 season or at 100 fps with a SONY® Cyber-shot RX10 III (SONY® Corporation) during the 2018 and 2019 seasons. Immediately after the bird had moved out of camera shot, a 1 m scale bar was carried into the frame and held directly over the trackways left by the bird in order to measure *U*. Pseudo-replication of the data was avoided by marking data location sites with GPS and using each location only once, as the Svalbard ptarmigan are highly territorial during the breeding season (Stokkan et al. 1986)*.*

From the 58 video recordings obtained, ${f}_{\mathrm{stride}}$ was calculated from 1 to 3 consecutive strides and ${l}_{\mathrm{stride}}$ derived by dividing *U* by ${f}_{\mathrm{stride}}$. ${t}_{\mathrm{stance}}$, ${t}_{\mathrm{swing}}$_,_ and *DF* were also calculated from the high-speed (100 fps) recordings. We determined the beginning of stance as immediately after foot was fully loaded on the ground, while the end of stance was marked immediately after the limb began protraction. From the high-speed recordings we also estimated the mechanical energy (the sum of the potential (*E*_p_) and kinetic (*E*_k_) energies) of the COM using the same method for male ptarmigans as described in Marmol-Guijarro et al. (2019). A mean body mass of 476.7 g for the females was obtained from literature (Lees et al. 2012a). Previous treadmill research by our group found that females are restricted to walking and grounded running gaits with a subsequent lower top speed (Lees et al. 2012a) than males, which can make use of an additional aerial running gait (Nudds et al. 2011; Marmol-Guijarro et al. 2019). Gaits were determined by the fluctuations in potential and kinetic energy of the COM (Cavagna et al. 1977). Grounded and aerial running were identified by the presence of an aerial phase (i.e. *DF* < 0.5). The analyses of the video recordings were conducted in Tracker® v.5.1.2 (Open Source Physics).

Prior to data analyses, all data points for each wild female ptarmigan were allocated to a specific gait based in phase relationship of *E*_p_ and *E*_k_ of the COM: in a walking gait *E*_p_ and *E*_k_ fluctuate out of phase, whilst in a grounded running gait *E*_p_ and *E*_k_ fluctuate in phase. Gait changes occur at *U* ranging from 0.85 to 0.95 ms^−1^. For analysis in this paper, the data from females moving on treadmill reported by Lees et al. (2012a) were placed into either a walking or grounded running gait based on the speed ranges of the wild females. We first analysed the kinematics of locomotion of female ptarmigan moving in situ over snowy ground and compared it to the treadmill data using linear models (LM). LM were then used to test whether limb kinematics within each gait changed in a similar way in both sexes (using male data from Lees et al. (2012a) and Marmol-Guijarro et al. (2019)) when moving over snowy substrates in situ using *U* as a covariate. Initially, both the slope and the intercept were tested for differences. The resulting model was then simplified by removing the interaction term (sex × *U*) if it was non-significant (i.e. similar slopes), and the LM was rerun to test only for differences in the intercepts. Shapiro-Wilks tests were used to ensure the residuals of the linear regressions and LMs approximated a normal distribution. To conform to the assumption of normally distributed data, in some cases kinematic parameters were transformed to log_10_.

Male ptarmigan are larger than females and differences in body mass and limb length are known to affect leg kinematics. Therefore, two sets of sex comparisons were done. The first set was analysed using the absolute value for each kinematic parameter regressed against *U*, where body mass and limb length effects were not considered. Then to account for size effects and test if male and female ptarmigan move in dynamically similar way, the second analysis used transformed kinematic parameters by relating them to hip height (*h*_hip_), taken from Lees et al. (2012a), and gravity (*g*), following Alexander and Jayes (1983): stride length $({\widehat{l}}_{\mathrm{stride}}={l}_{\mathrm{stride}}/{h}_{\mathrm{hip}})$, stride frequency (${\widehat{f}}_{\mathrm{stride}}={f}_{\mathrm{stride}}/\sqrt{g/{h}_{\mathrm{hip}}}$), stance (${\widehat{t}}_{\mathrm{stance}}={t}_{\mathrm{stance}}/\sqrt{{h}_{\mathrm{hip}}/g}$) and swing (${\widehat{t}}_{\mathrm{swing}}={t}_{\mathrm{swing}}/\sqrt{{h}_{\mathrm{hip}}/g}$), and *Û* ($U/\sqrt{{h}_{\mathrm{hip}}\times g}$) as the speed covariate. *DF* is dimensionless and was therefore not corrected when regressed against *Û*. All the statistical analyses were conducted in R v. 3.6.6 “Holding the Windsock” (R Core Team 2020) and the results are summarised in Tables 1, 2 and Fig. S1.

**Table 1:** Results of the linear models investigating differences in the absolute kinematics between female ptarmigan locomoting on treadmills and in the field

Gait	Parameter	Final model	*R*^2^	Equations
Walking	${l}_{\mathrm{stride}}$	*U* (*F*_1, 24_ = 81.769; *P* < 0.001)	0.764	♂ & ♀ = 0.214 *U* + 0.115
	${f}_{\mathrm{stride}}$	*U* (*F*_1, 24_ = 73.663; *P* < 0.001)	0.744	♂ & ♀ = 2.041 *U* + 1.193
	${{\mathrm{log}}_{10}t}_{\mathrm{stance}}$	Log_10_ *U* (*F*_1, 18_ = 75.568; *P* < 0.001)	0.797	♂ & ♀ = 0.200 *U* ^−0.564^
	${{\mathrm{log}}_{10}t}_{\mathrm{swing}}$	Log_10_ *U* (*F*_1, 16_ = 7.576; *P* < 0.0001) sex (*F*_1, 16_ = 5.650; *P* < 0.05) Log_10_ *U* × sex (*F*_1, 16_ = 4.851; *P* < 0.05)	0.575	♂ = 0.133 *U* ^−0.098^ ♀ = 0.127 *U* ^−0.332^
	Duty Factor	*U* (*F*_1, 16_ = 35.36; *P* < 0.01) sex (*F*_1, 16_ = 12.937; *P* < 0.01) *U* × sex (*F*_1, 16_ = 6.891; *P* < 0.05)	0.733	♂ = − 0.207 *U* + 0.792 ♀ = − 0.075 *U* + 0.682
Grounded running	${l}_{\mathrm{stride}}$	*U* (*F*_1, 34_ = 55.191; *P* < 0.001)	0.608	♂ & ♀ = 0.157 *U* + 0.164
	${f}_{\mathrm{stride}}$	*U* (*F*_1, 34_ = 40.303; *P* < 0.001)	0.529	♂ & ♀ = 1.297 *U* + 1.839
	${t}_{\mathrm{stance}}$	*U* (*F*_1, 24_ = 55.484; *P* < 0.001)	0.686	♂ & ♀ = − 0.108 *U* + 0.296
	${{\mathrm{log}}_{10}t}_{\mathrm{swing}}$	Log_10_ *U* (*F*_1, 24_ = 7.0584; *P* < 0.05)	0.195	♂ & ♀ = 0.140 *U* ^−0.213^
	Duty Factor	*U* (*F*_1, 24_ = 21.409; *P* < 0.001)	0.449	♂ & ♀ = − 0.113 *U* + 0.682

**Table 2:** Results of the linear models investigating differences in kinematics between male and female ptarmigan

Gait	Parameter	Final model	*R*^2^	Equations
*Kinematic comparison with absolute values*				
Walking	${l}_{\mathrm{stride}}$	*U* (*F*_1, 67_ = 137.419; *P* < 0.001) sex (*F*_1, 67_ = 10.736; *P* < 0.001)	0.689	♂: = 0.202 *U* + 0.143 ♀: = 0.202 *U* + 0.123
	${f}_{\mathrm{stride}}$	*U* (*F*_1, 67_ = 149.940; *P* < 0.001) sex (*F*_1, 67_ = 11.125; *P* < 0.01)	0.698	♂: = 2.036 *U* + 1.032 ♀: = 2.036 *U* + 1.205
	${{\mathrm{log}}_{10}t}_{\mathrm{stance}}$	Log_10_ *U* (*F*_1, 39_ = 140.057; *P* < 0.001) sex (*F*_1, 39_ = 34.525; *P* < 0.001)	0.808	♂: = 0.218 *U* ^−0.647^ ♀: = 0.190 *U* ^−0.647^
	${{\mathrm{log}}_{10}t}_{\mathrm{swing}}$	*U* (*F*_1, 40_ = 14.349; *P* < 0.001)	0.286	♂ & ♀ = 0.131 *U* ^−0.234^
	Duty Factor	*U* (*F*_1, 39_ = 42.223; *P* < 0.001) sex (*F*_1, 39_ = 34.947; *P* < 0.001) *U* × sex (*F*_1, 39_ = 10.611; *P* < 0.01)	0.674	♂: = − 0.230 *U* + 0.814 ♀: = − 0.075 *U* + 0.682
Grounded running	${l}_{\mathrm{stride}}$	*U* (*F*_1, 84_ = 58.446; *P* < 0.001) sex (*F*_1, 84_ = 11.127; *P* < 0.01) *U* × sex (*F*_1, 84_ = 4.869; *P* < 0.05)	0.451	♂: = 0.346 *U* ^0.291^ ♀: = 0.318 *U* ^0.532^
	${f}_{\mathrm{stride}}$	*U* (*F*_1, 84_ = 117.949; *P* < 0.001) sex (*F*_1, 84_ = 2.269; *P* = 0.136) *U* × sex (*F*_1, 84_ = 4.358; *P* < 0.05)	0.583	♂: = 1.925 *U* + 0.979 ♀: = 1.309 *U* + 1.851
	${{\mathrm{log}}_{10}t}_{\mathrm{stance}}$	Log_10_ *U* (*F*_1, 50_ = 130.3; *P* < 0.001)	0.717	♂ & ♀ = 0.189 *U* ^−0.872^
	${{\mathrm{log}}_{10}t}_{\mathrm{swing}}$	Log_10_ *U* (*F*_1, 50_ = 17.883; *P* < 0.001)	0.249	♂ & ♀ = 0.144 *U* ^−0.302^
	Duty Factor	*U* (*F*_1, 50_ = 33.96; *P* < 0.001)	0.393	♂ & ♀ = − 0.111 *U* + 0.677
Body size (body mass and limb length)-corrected kinematic comparison				
Walking	${\widehat{l}}_{\mathrm{stride}}$	*Û* (*F*_1, 68_ = 145.18; *P* < 0.001)	0.676	♂ & ♀ = 1.571 *Û* + 0.810
	${\widehat{f}}_{\mathrm{stride}}$	*Û* (*F*_1, 68_ = 147.39; *P* < 0.001)	0.679	♂ & ♀ = 0.332 *Û* + 0.144
	${\mathrm{log}}_{10}{\widehat{t}}_{\mathrm{stance}}$	Log_10_ *Û* (*F*_1, 40_ = 153.83; *P* < 0.001)	0.789	♂ & ♀ = 1.351 *Û* ^−0.662^
	${\mathrm{log}}_{10}{\widehat{t}}_{\mathrm{swing}}$	Log_10_ *Û* (*F*_1, 38_ = 19.028; *P* < 0.001) sex (*F*_1, 38_ = 6.343; *P* < 0.05)	0.363	♂: = 0.901 *Û* ^−0.245^ ♀: = 1.016 *Û* ^−0.245^
	Duty Factor	*Û* (*F*_1, 37_ = 40.928; *P* < 0.001) sex (*F*_1, 37_ = 34.947; *P* < 0.001) *Û* × sex (*F*_1, 37_ = 11.907; *P* < 0.01)	0.674	♂: = − 0.299 *Û* + 0.814 ♀: = − 0.092 *Û* + 0.682
Grounded running	${\mathrm{log}}_{10}{\widehat{l}}_{stride}$	Log_10_ *Û* (*F*_1, 84_ = 58.446; *P* < 0.001) sex (*F*_1, 84_ = 8.539; *P* < 0.05) Log_10_ *Û* × sex (*F*_1, 84_ = 4.869; *P* < 0.05)	0.441	♂: = 2.164 *Û* ^0.291^ ♀: = 2.231 *Û* ^0.532^
	${\widehat{f}}_{\mathrm{stride}}$	*Û* (*F*_1, 84_ = 116.187; *P* < 0.001) sex (*F*_1, 84_ = 3.098; *P* = 0.082) *Û* × sex (*F*_1, 84_ = 7.456; *P* < 0.01)	0.587	♂: = 0.333 *Û* + 0.130 ♀: = 0.200 *Û* + 0.231
	${\mathrm{log}}_{10}{\widehat{t}}_{\mathrm{stance}}$	Log_10_ *Û* (*F*_1, 49_ = 108.936; *P* < 0.001) sex (*F*_1, 49_ = 1.374; *P* = 0.247)	0.680	♂: = − 1.168 *Û* + 2.333 ♀: = − 1.168 *Û* + 2.468
	${\mathrm{log}}_{10}{\widehat{t}}_{\mathrm{swing}}$	Log_10_ *Û* (*F*_1, 49_ = 17.494; *P* < 0.001) sex (*F*_1, 49_ = 2.809; *P* < 0.05)	0.264	♂: = 1.005 *Û* ^−0.300^ ♀: = 1.071 *Û* ^−0.300^
	Duty Factor	*Û* (*F*_1, 50_ = 31.456; *P* < 0.001)	0.374	♂ & ♀ = 0.669 *Û* − 0.133

## Results

### Comparison of absolute values for wild and treadmill locomotion kinematics in females

**Fig. 1:**
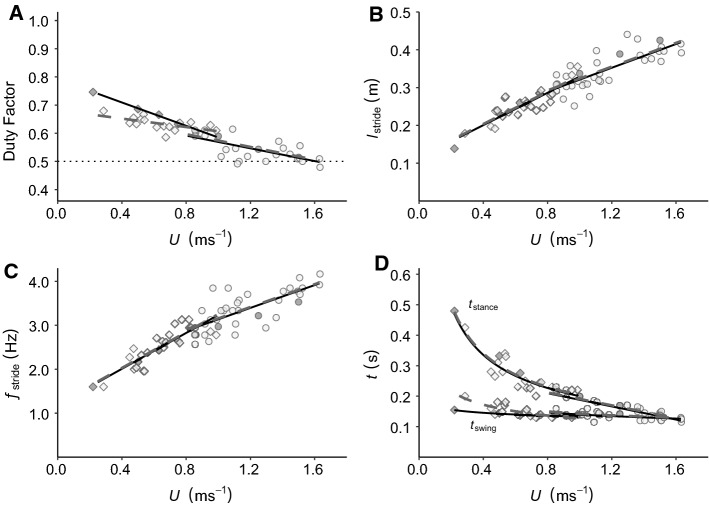
Female kinematic parameters plotted against speed (*U*). *DF* (**a**), ${l}_{\mathrm{stride}}$ (**b**), ${f}_{\mathrm{stride}}$ (**c**) and ${t}_{\mathrm{stance}}$ and ${t}_{\mathrm{swing}}$ (**d**) are plotted against *U* for females moving in the wild (dashed line and white points) compared to females moving on treadmills (solid line and grey points) during walking (rhomboids) and grounded running (circles). The results of the linear models with the best-fine line equations are summarised in Table 1

When compared to the previous treadmill studies (Lees et al. 2012a) females did have a higher top speed in the wild, increasing by up to 8% to a maximum of 1.63 ms^−1^ (Fig. 1) but in line with treadmill studies they never displayed an aerial running gait (Fig. 1a). Overall locomotor kinematics for the female ptarmigan are similar in both walking and grounded running gaits when moving under laboratory and field conditions.

Within the walking gait, ${l}_{\mathrm{stride}}$ and ${f}_{\mathrm{stride}}$ increased linearly with increasing *U* (Fig. 1b and c; Table 1). ${t}_{\mathrm{stance}}$ decreased curvilinearly with *U* and *DF* decreased linearly with *U* (Fig. 1a and d; Table 1)*.* The only difference between the two groups was that ${t}_{\mathrm{swing}}$ was slightly higher in females walking in the wild at the slowest speeds and decreased at a faster rate with *U* than in females walking on treadmills (Fig. 1d; Table 1).

For grounded running, ${l}_{\mathrm{stride}}$ and ${f}_{\mathrm{stride}}$ increased linearly with *U* (Fig. 1b and c; Table 1)*.* ${t}_{\mathrm{stance}}$ and ${t}_{\mathrm{swing}}$ decreased linearly and curvilinearly with increasing *U*, respectively (Fig. 1d; Table 1). Similar to the walking gait, *DF* decreased linearly with *U* (Fig. 1a; Table 1).

### Comparison of absolute values for wild kinematics between females and males

**Fig. 2:**
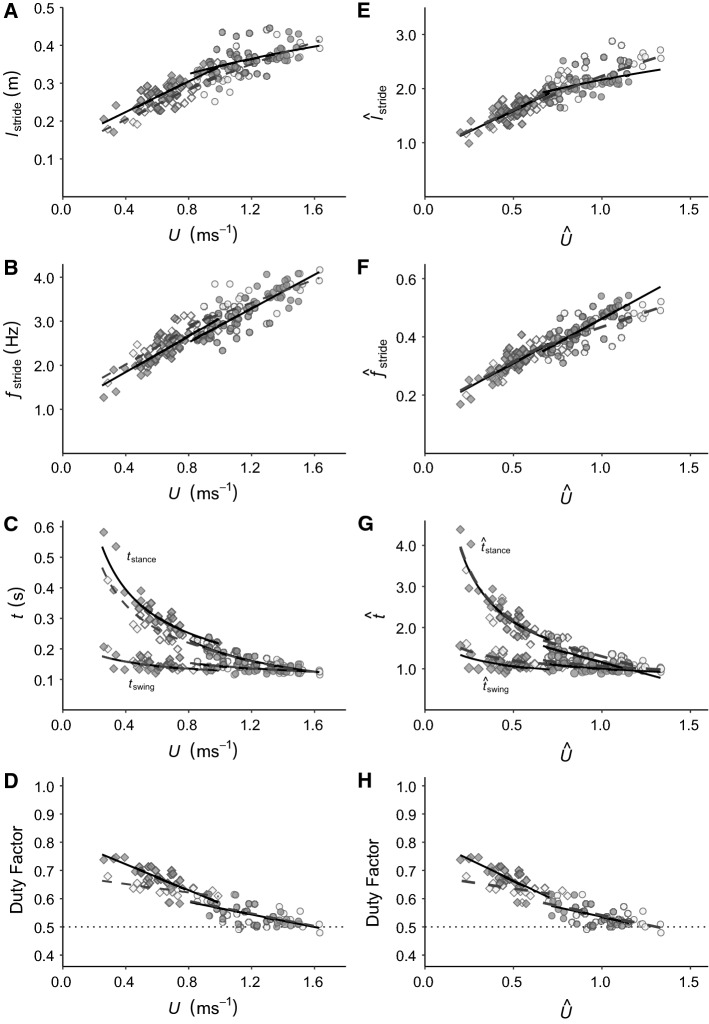
Kinematic parameters plotted against speed (*U*) and relative speed (*Û*), compensated for body size (body mass and limb length), for each gait and both sexes*.* ${l}_{\mathrm{stride}}$ (**a**), ${f}_{\mathrm{stride}}$ (**b**), ${t}_{\mathrm{stance}}$ and ${t}_{\mathrm{swing}}$ (**c**) Duty Factor (**d**) and the body-size-compensated correlates (please see text for definitions), ${\widehat{l}}_{\mathrm{stride}}$ (**e**), ${\widehat{f}}_{\mathrm{stride}}$ (**f**), ${\widehat{t}}_{\mathrm{stance}}$ and ${\widehat{t}}_{\mathrm{swing}}$ (**g**) and Duty Factor (**g**) are plotted against *U* (left column) and *Û* (right column), respectively, for females (dashed line and white points) and males (solid line and grey points) when walking (rhomboids) and grounded running (circles) in situ over snowy substrate. Male data from. The results of the linear models with the best-fine line equations are summarised in Table 2

For the walking gait, ${l}_{\mathrm{stride}}$ and ${f}_{\mathrm{stride}}$ increased linearly with increasing *U* at the same rate in males and females. However, ${l}_{\mathrm{stride}}$ and ${f}_{\mathrm{stride}}$ were lower (14%) and higher (16%) across all walking speeds in females (Fig. 2a and b; Table 2), respectively. With increasing *U*, ${t}_{\mathrm{stance}}$ and ${t}_{\mathrm{swing}}$ decreased curvilinearly at the same rate for both sexes. ${t}_{\mathrm{stance}}$ was shorter across all speeds in females; however, ${t}_{\mathrm{swing}}$ was similar for both sexes at all speeds (Fig. 2c; Table 2). Consequently, *DF* is lower in females at the slowest speeds relatively to the *DF* of males but becomes similar at the fastest walking speeds for both sexes (Fig. 2d; Table 2).

Within the grounded running gait, females had a lower ${l}_{\mathrm{stride}}$ at the slowest grounded running *U* that progressively became similar to the ones observed in the males as they moved faster. The opposite was true for ${f}_{\mathrm{stride}}$, where females took faster strides at the slowest grounded running *U,* that became comparable to the ${f}_{\mathrm{stride}}$ values observed in males as they moved faster (Fig. 2a and b; Table 2). ${t}_{\mathrm{stance}}$ and ${t}_{\mathrm{swing}}$ decreased curvilinearly with increasing *U* and was similar across all values of *U* for both sexes (Fig. 2c; Table 2). Therefore, *DF* was also similar at all speeds in both sexes (Fig. 2d; Table 2).

### Body-size-corrected kinematics of wild females and wild males

After accounting for body mass and limb length, most of the differences between sexes reported above during the walking gait disappeared, whereas differences in ${\widehat{t}}_{\mathrm{swing}}$ became apparent. ${\widehat{l}}_{\mathrm{stride}}$ and ${\widehat{f}}_{\mathrm{stride}}$ increased linearly with, and were similar at all values of, *Û* for males and females (Fig. 2e and f; Table 2). ${\widehat{t}}_{\mathrm{stance}}$ decreased curvilinearly with *Û*, and again no differences were detected between the sexes (Fig. 2g, Table 2). ${\widehat{t}}_{\mathrm{swing}}$ decreased curvilinearly with *Û* at the same rate for each sex. Across all *Û*, however, females had a greater ${\widehat{t}}_{\mathrm{swing}}$ (Fig. 2g, Table 2). Concordant with the non-size-corrected results above, females had a lower *DF* at lower *Û* than the males, but their *DF* converged at the highest *Û* (Fig. 2h, Table 2). Thus, the differences in ${l}_{\mathrm{stride}}$, ${f}_{\mathrm{stride}}$ and ${t}_{\mathrm{stance}}$, could be attributed to the unequal body mass and limb lengths of males and females, because those differences disappeared after size correction.

In contrast to the walking gait data, all sex differences identified in the relationships between the absolute kinematic parameters and *U* within a grounded running were conserved after accounting for the effects of body mass and limb length. ${\widehat{l}}_{\mathrm{stride}}$ increased curvilinearly with *Û* and was longer in males at lower *Û*, but, conversely, longer in females at higher *Û* (Fig. 2e; Table 2). ${\widehat{f}}_{\mathrm{stride}}$ increased with *Û*. Males had a higher ${\widehat{f}}_{\mathrm{stride}}$ at higher *Û* than females, but their ${\widehat{f}}_{\mathrm{stride}}$ converged at the lowest grounded running gait *Û* (Fig. 2f, Table 2). ${\widehat{t}}_{\mathrm{stance}}$ and ${\widehat{t}}_{\mathrm{swing}}$ decreased in a curvilinear manner with increasing *Û*, and decreased at a similar rate in males and females. Females, however, had greater ${\widehat{t}}_{\mathrm{stance}}$ and ${\widehat{t}}_{\mathrm{swing}}$ than the males across all *Û* (Fig. 2g; Table 2). Despite these differences, *DF* was similar in each sex and decreased linearly with increasing *Û* in both (Fig. 2h, Table 2). In contrast to the sex differences in the absolute values for the walking gait, body size cannot explain the kinematic differences in grounded running between male and female ptarmigan.

## Discussion

Generally, walking in females, aside from ${t}_{\mathrm{swing}}$, was not greatly affected by moving over snow relative to on a treadmill (Fig. 1). The robustness of a walking gait over snow was found previously in males (Marmol-Guijarro et al. 2019) where an increased stability is achieved via passive mechanics of the musculoskeletal system (Jindrich and Full 2002) in tandem with the presence of a period of double support, thereby contributing to lower lateral displacements of the centre of mass (COM) after by a medio lateral perturbations compared to a running gait (Qiao et al. 2012; Qiao and Jindrich 2014). The greater absolute ${t}_{\mathrm{swing}}$ observed in the wild females at very slow walking *U*, compared to females on treadmills, could be caused by a reduction in the toe clearance. Toe dragging was observed in the trackways associated with very slow, which would increase the length of time it took to swing the limb (see Fig. S1), although snow hardness and/or depth may also be important. Toe dragging may be the best option, as increasing toe clearance by lifting the foot clear of the snow during each stride could potentially impose greater energetic penalties for each stride (Gates et al. 2012; Wu and Kuo 2016). The absence of an effect on ${t}_{\mathrm{swing}}$ at faster speeds likely reflects the fact that toe dragging is not an option when moving faster as the risk of falling, limb failure or injury becomes too great (Schulz 2011; Gates et al. 2012).

Interestingly, and unlike the findings for male ptarmigan (Marmol-Guijarro et al. 2019), there were no differences in the kinematics of a grounded running gait for females in the wild when compared to the treadmill. This result was surprising as faster gaits are usually more susceptible to destabilisation of the COM because of the relatively greater time spent with only one limb on the ground (Qiao et al. 2012; Qiao and Jindrich 2014), particularly when moving faster over a highly changeable substrate like snow that can vary from icy to soft. Birds minimise the risk of falling by a reduction of ${l}_{\mathrm{stride}}$ and an increase in ${f}_{\mathrm{stride}}$_,_ achieved by an earlier retraction of the limb just before the stance phase (Bhatt et al. 2005; Daley et al. 2006; Daley and Biewener 2006; Cappellini et al. 2010; Clark and Higham 2011; Birn-Jeffery and Daley 2012), that ensures that the COM passes over the supporting limb (Seyfarth et al. 2003). Similar adjustments have also been reported in a number of avian species when negotiating obstacles and/or drops, including the guinea fowl (*Numida meleagris*) (Daley et al. 2006; Daley and Biewener 2006; Birn-Jeffery et al. 2014), the pheasant (*Phasianus colchinus*) (Birn-Jeffery and Daley 2012; Birn-Jeffery et al. 2014), the bobwhite (*Colinus virginianus*), the turkey (*Meleagris gallopavo*) and ostriches (*Struthio camelus*) (Birn-Jeffery et al. 2014) or when locomotion occurs over other slippery surfaces as shown in the guinea fowl (Clark and Higham 2011) and in humans (Bhatt et al. 2005; Cappellini et al. 2010). The Svalbard ptarmigan males are thought to adjust their limb kinematics in this way to negotiate with snowy substrate while using grounded running (Marmol-Guijarro et al. 2019). Generally, grounded running is thought to improve locomotor stability due to longer supporting phases (Gatesy and Biewener 1991; Andrada et al. 2013), while still recovering energy through the elastic elements of the limb (Rubenson et al. 2004; Nudds et al. 2011). Grounded running is also associated with other benefits like an increased stability of the head (Hancock et al. 2007) and a reduction in the bouncing of non-locomotor tissues (Daley and Usherwood 2010). It is puzzling therefore as to why the female ptarmigan are not modifying their limb kinematics during grounded running over snow when all these potential effects could be beneficial. We believe it is likely that the differential influences of snow on a grounded running gait for males and females can be explained by differences in limb compliance. If female ptarmigan had a more compliant limb during grounded running relative to males, this may explain why their kinematics were less affected at increasing speeds over an inherently unstable substrate. Therefore, by examining sex differences between the kinematics of walking and grounded running it should be possible to detect whether any disparities are caused solely by sexual dimorphism in body mass and limb length or if the apparent robustness of female locomotion over snow is explained by differences in limb posture.

### Sex differences in locomotion over snow

Males are up to 6% heavier and have longer limbs (Steen and Unander 1985; Lees et al. 2012a) than females. Therefore, if the two sexes are to move in a dynamically similar way and the absolute kinematics and hip heights scale with body mass and limb length (Alexander and Jayes 1983), the rates of change in the kinematic parameters with increasing *U* should remain similar. Except for ${t}_{\mathrm{swing}}$ and *DF*, all the absolute kinematic parameters during a walking gait in male and female ptarmigan are in line with the differences associated with body size for birds (Gatesy and Biewener 1991; Abourachid and Renous 2000). The smaller females had smaller absolute ${l}_{\mathrm{stride}}$, and greater absolute ${f}_{\mathrm{stride}}$ and absolute ${t}_{\mathrm{stance}}$ at any given *U* compared to the males (Fig. 2a–c). The differences in the absolute ${l}_{\mathrm{stride}}$, ${f}_{\mathrm{stride}}$ and ${t}_{\mathrm{stance}}$, however, all disappeared when comparisons were made using body mass and limb length-corrected values for any given relative speed *Û* (Fig. 2e–g). Therefore, male and female ptarmigan are using a dynamic similar walking gait where body size has a major influence on the kinematic parameters.

Conversely, male and female ptarmigan do not appear to move in a dynamically similar way within a grounded running gait. As the differences in the absolute ${l}_{\mathrm{stride}}$ and absolute ${f}_{\mathrm{stride}}$ between males and females when using a grounded running gait persisted after correcting for body size (body mass and limb length), a difference appeared in size-corrected ${\widehat{t}}_{\mathrm{stance}}$. Differences, such as these, in limb posture are often associated with a departure from dynamic similarity (Gatesy and Biewener 1991; Alexander and Jayes 1983). The greater ${\widehat{l}}_{\mathrm{stride}}$ of the females at the top end of the speed range during grounded running, may be attributed to a greater excursion of a more crouched limb in females relative to males (Fig. 2e). Changes in limb posture may also explain the lower ${\widehat{f}}_{\mathrm{stride}}$ of the females for almost all the range of grounded running *Û* (Fig. 2f). A more crouched posture of the females is also supported by the greater ${\widehat{t}}_{\mathrm{stance}}$ across grounded running *Û* (Gatesy and Biewener 1991) (Fig. 2g). Adjusting their ${\widehat{t}}_{\mathrm{stance}}$ would confer additional stability to females (Gatesy and Biewener 1991; Andrada et al. 2013), which might explain why they do not change their kinematic parameters in the same way as males do when moving on snow.

The relatively longer ${\widehat{t}}_{\mathrm{swing}}$ of the female ptarmigan for all walking and grounded running *Û* (Fig. 2g) can likely be explained by departures in the mass proportions of each of the limb segments when compared to ptarmigan males. Experiments on limb loading in birds demonstrate that if the distal portion is heavier relative to the proximal portion, for example with loads added equivalent to 5% of their body mass, then these birds face difficulties in decelerating the swinging limb as the extra load caused greater moments of inertia, increasing ${t}_{\mathrm{swing}}$(Tickle et al. 2010). Similarly, a study of three species of shore birds revealed that ${\widehat{t}}_{\mathrm{swing}}$ was longer in the species that naturally possessed limbs with heavier distal portions (Kilbourne et al. 2016). In the case of the female ptarmigan, the longer ${\widehat{t}}_{\mathrm{swing}}$ might be explained by departures in mass of the proximal limb segments (the femoral and the tibiotarsal regions) with females having smaller muscles, relative to the males. These differences in muscle mass would make it more challenging for the females to counter the moments of inertia of the swinging limb, if the mass of the distal limb segment in both sexes is proportional. Yet, data on joint kinematics in the ptarmigan are needed to test this hypothesis.

Sexual dimorphism in limb posture is evident in leghorn chicken, where females have a more upright posture compared to the males (Rose et al. 2015, 2016b). In leghorns the more upright limbs reduce the work that muscles must produce and bones must resist due to a proportionally greater visceral mass of females compared to males, without decreasing ${t}_{\mathrm{stance}}$ (Rose et al. 2016b). However, this is the opposite of what we see in the female ptarmigan, which have a more crouched posture compared to the males. These differences are likely explained by important differences between how female leghorns and ptarmigan move. In contrast with female ptarmigan, the female leghorns are not capable of grounded running, at least on treadmills, and all the sex differences in their locomotor kinematics were found during a walking gait (Rose et al. 2016b). Moreover, female leghorns have been artificially selected to be in a permanently gravid state able to lay eggs all year (Mitchell et al. 1931; Rose et al. 2016b), whereas female ptarmigan breed once a year normally in June (Stokkan et al. 1986). Therefore, female leghorns must support a 1.32 times larger reproductive organ mass for longer; maximum ovaries mass in the female ptarmigan is 34.60 g in June (Mitchell et al. 1931; Stokkan et al. 1986). Conversely, the ptarmigan females in this study were sampled at the end of April/beginning of May, at the onset of ovarian development prior egg laying (Stokkan et al. 1986). As a result, the female ptarmigan in this study were free from the constraint of having to support any extra weight, which means that joint moments can be reduced enough to make crouched postures attainable, thus allowing for longer stance periods, lower power demands (Usherwood 2013) and greater locomotor stability. Furthermore, the risks of bone fracture in ptarmigan females might be lower, as bone calcium deficits are expected to be negligible at this time of the year. Bone fracture is a particular risk for Leghorn hens as they often suffer from osteoporosis as a result of the permanent transfer of calcium to the egg shell (Whitehead 2004).

### Implications for limb architecture and muscle anatomy

From an anatomical perspective, the limbs of the female ptarmigan may not be as suited to generating force in the way males do in order to run faster. The PCSA of a muscle is directly related to the forces that muscles, in this case of the pelvic limbs, can produce and depends directly on the mass and volume of the muscle and the length and angle of pennation of the fascicles within that muscle. Often in birds, males have a greater PCSA on several locomotor muscles, including the ilitibialis cranialis and lateralis, the ilifibularis, and the flexor cruris medialis and lateralis pars pelvica, at the onset of maturity compared to females, conferring them an improved locomotor performance (Rose et al. 2016b, 2016c). The Svalbard ptarmigan appear to fit this general pattern with a higher *U* achieved by males under both treadmill and wild conditions (Lees et al. 2012a). Higher *U* are likely due to a greater PSCA and more upright posture of the males. It is particularly interesting that wild females’ top speed only increased by 8% (from 1.5 to 1.62 ms^−1^) compared to the speed achieved running on treadmills (Fig. 1), whereas in males the differences are greater for the wild individuals that could achieve up to a 34% increase in top speed (from 2 to 2.76 ms^−1^) (Marmol-Guijarro et al. 2019). These differences between females and males may be related to the relative composition of fibre types in the ptarmigan limb muscles. Indeed, sex differences in fibre type composition are common in many muscles of a number of species. For example, in the mouse hindlimb the expression levels of the myosin heavy chain isoform for type-IIB fibres (fast glycolytic) are higher in females compared to males in the soleus (64% in females vs. 42% in males) and the tibialis lateralis muscles (75% in females vs. 61% in males), whilst in the plantaris muscle higher levels of the type-IIB isoform are observed in males (84%) than in females (63%) (Haizlip et al. 2015). Similarly in humans, the vastus lateralis muscle located in the thigh have higher percentages of type-I myosin isoforms (slow oxidative) in females (41%) than in males (34%), while type-IIA isoform is higher in males (46%) than in females (36%) (Staron et al. 2000). In the tropical golden collared manakin (*Manacus vitellinus*), 49% of the fibres in the scapulohumeralis caudalis and 47% of the fibres in the supracoracoides in the males correspond to fast oxidative glycolytic and fast oxidative fibres compared to only 4% and 2% in the females, respectively; as these muscles are key for the wing snaping behaviour of males during courtship (Schultz et al. 2001). Similar to the manakin, sexual selection favouring higher proportions of fast twitch fibres on the hindlimb muscles of the ptarmigan males provides further support to the improved locomotor capacity show by them compared to females. On the other hand, a greater proportion of slow oxidative fibres would explain the greater ${\widehat{t}}_{\mathrm{stance}}$ observed for the during grounded running in females, as they would find challenging to produce enough force to move faster and restricting them from using aerial gaits.

## Future directions

There is a marked seasonal variation in body mass in the Svalbard rock ptarmigan over the year (Steen and Unander 1985; Stokkan et al. 1986). In particular for females, it would be interesting to study their locomotion throughout breeding cycle, in particular when they are at their heaviest prior to egg laying, and throughout winter, when they double their body weight seasonally to further test some of these hypotheses. Sampling across the range of seasonal mass fluctuations would enable us to tease apart the influence of mass gain specifically associated with reproductive effort and that associated with overwintering survival. These data would be particularly interesting in the Svalbard ptarmigan as males have adaptations that mitigate the influence of winter mass gain, meaning they move more efficiently in terms of the metabolic cost of locomotion, albeit over a reduced speed range (Lees et al. 2010). It is unknown if females show the same adaptations. At the muscular level, a detailed analysis of the muscle architecture of the limb, including PSCA measures, fibre typing and limb proportions, of the male and the female ptarmigan remains to be investigated.

Comparisons between laboratory and field datasets are challenging. Snow by its own nature is a substrate where a number of physical properties relevant for terrestrial locomotion, including snow harness, humidity, stratification and depth, might change at very short spatiotemporal scale. Moreover, studying the wild ptarmigan locomotor kinematics through video recordings depends on the ability to identify temporal events (i.e. ${t}_{\mathrm{stance}}$) that can be hindered either because foot landing and lifting may occur in deep snow or simply because the frame rate used for recording may not precisely capture such event. This results in estimation errors particularly affecting $DF$ (Fig. S2). With the development of smaller and more powerful batteries, bio-loggers that allow the study of key ecological, behavioural and physiological aspects of wild birds may be used to link these traits to adaptations in the locomotor system. For example, bio-loggers that are capable of detecting the time that the GRF acts on the feet may give a more accurate detection of foot landing and take-off. These types of loggers have been already used to analyse the locomotor biomechanics in other species including ostriches (Daley et al. 2016), cheetah (Wilson et al. 2013) and elephants (Ren and Hutchinson 2008). Moreover, they could provide valuable information regarding underlying evolutionary constraints leading to disparate locomotor capabilities in males and females in terms of locomotor biomechanics and energetics. Overall, our study adds new data on sex differences in locomotion and the kinematic mechanisms used by birds to improve stability while moving over snow.

## Supplementary Information

Below is the link to the electronic supplementary material.

Supplementary file1 (DOCX 3975 KB)

## Data Availability

The raw dataset generated during this project is available via the FigShare repository (https://doi.org/10.6084/m9.figshare.12205247).
