# Supplementary material for: Does posture explain the kinematic differences in a grounded running gait between male and female Svalbard rock ptarmigan (Lagopus muta hyperborea) moving on snow?
Source: Polar Biol. 2021 May 5;44(6):1141–52. doi: 10.1007/s00300-021-02872-x (PMC8550507; doi:10.1007/s00300-021-02872-x)
Supplement: Supplementary file 1 — Supplementary file1 (DOCX 3975 KB) [file 300_2021_2872_MOESM1_ESM.docx]

Electronic Supplementary Material

Does posture explain the kinematic differences in a grounded running gait between male and female Svalbard rock ptarmigan (*Lagopus muta hyperborea*) moving on snow?

Polar Biology

Andres Marmol-Guijarro^1^, Robert Nudds^1^, Lars Folkow^2^, John Lees^3^ & Jonathan Codd^1*^

^1^School of Biological Sciences, Faculty of Biology, Medicine & Health, University of Manchester, Manchester, UK. ^2^Department of Arctic and Marine Biology, University of Tromsø - the Arctic University of Norway, Tromsø, Norway. ^3^Department of Physics, Chemistry and Biology, Linköpings Universitet, Linköping, Östergötland, Sweden.

*Author for Correspondence:

Dr Jonathan Codd

e-mail: [jonathan.codd@manchester.ac.uk](mailto:jonathan.codd@manchester.ac.uk)

**Fig. S1**. Examples of tracks highlighting incidences of toe dragging for female ptarmigan moving soft snow at very slow (a, *U* = 0.5 ms^-1^) and faster (b and c, *U* = 0.68 & 1.5 ms^-1^ respectively) locomotion speeds. Toe dragging in snow may decelerate the swinging limb, thus increasing $\boldsymbol{t}_{\mathbf{swing}}$*.* Toe dragging decreases with increasing *U.*


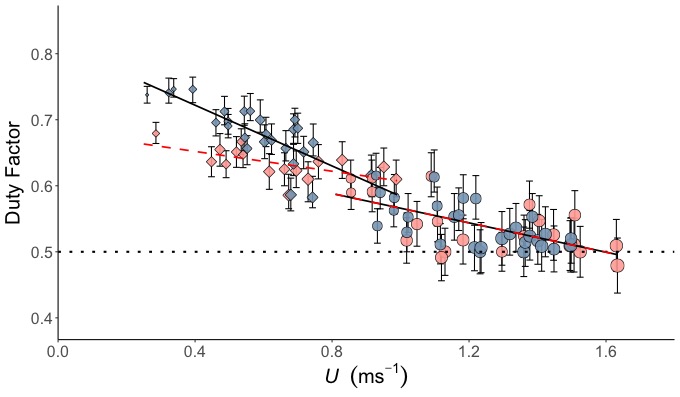


**Fig. S2.** Precision-related issues in Duty Factor (*DF*) estimates. *DF* is plotted against speed (*U*) for females (red points and dashed red lines) and males (blue points and solid lines) while walking (rhomboids) and grounded running (circles) over snow. The relative size of each point represents the error estimates in *DF*, the larger being associated with larger errors. The error bars represent the upper and lower limits if the beginning or the ending of $t_{\mathrm{stance}}$ was delayed by one hundredth of a second (one frame). All data points and limits were obtained from videos recorded at 100 fps using the same manual technique described in the method section of the paper. *DF* lower than 0.5 (0.48 and 0.49, respectively) were recorded in two females and would technically be classified to an aerial running gait. However, at these *U*, $t_{\mathrm{stance}}$ and $t_{\mathrm{swing}}$ vary only in a few hundredths of second, which is at the limit of the accuracy that can be obtained from the video recordings. For example, if the foot lifting of these two females occurs only one hundredth of a second (a frame) later, *DF* would become 0.52 as suggested by the upper limits of the error bars and would be reassigned to a grounded running gait. Determining foot landing or lifting when the bird’s feet sink into the substrate can be challenging.
